# Effect of a Default Order vs an Alert in the Electronic Health Record on Hepatitis C Virus Screening Among Hospitalized Patients: A Stepped-Wedge Randomized Clinical Trial

**DOI:** 10.1001/jamanetworkopen.2022.2427

**Published:** 2022-03-17

**Authors:** Shivan J. Mehta, Jessie Torgersen, Dylan S. Small, Colleen P. Mallozzi, John D. McGreevey, Charles A.L. Rareshide, Chalanda N. Evans, Mika Epps, David Stabile, Christopher K. Snider, Mitesh S. Patel

**Affiliations:** 1 Department of Medicine, Perelman School of Medicine, University of Pennsylvania, Philadelphia; 2 Center for Health Care Innovation, University of Pennsylvania, Philadelphia; 3 The Wharton School, University of Pennsylvania, Philadelphia; 4 University of Pennsylvania Health System, University of Pennsylvania, Philadelphia; 5 Center for Applied Health Informatics, University of Pennsylvania Health System, Philadelphia; 6 Penn Medicine Nudge Unit, University of Pennsylvania, Philadelphia; 7 Crescenz Veterans Affairs Medical Center, Philadelphia, Pennsylvania; 8 Ascension Health, St Louis, Missouri

## Abstract

**Importance:**

Hepatitis C virus (HCV) screening has been recommended for patients born between 1945 and 1965, but rates remain low.

**Objective:**

To evaluate whether a default order within the admission order set increases HCV screening compared with a preexisting alert within the electronic health record.

**Design, Setting, and Participants:**

This stepped-wedge randomized clinical trial was conducted from June 23, 2020, to April 10, 2021, at 2 hospitals within an academic medical center. Hospitalized patients born between 1945 and 1965 with no history of screening were included in the analysis.

**Interventions:**

During wedge 1 (a preintervention period), both hospital sites had an electronic alert prompting clinicians to consider HCV screening. During wedge 2, the first intervention wedge, the hospital site randomized to intervention (hospital B) had a default order for HCV screening implemented within the admission order set. During wedge 3, the second intervention wedge, the hospital site randomized to control (hospital A) had the default order set implemented.

**Main Outcomes and Measures:**

Percentage of eligible patients who received HCV screening during the hospital stay.

**Results:**

The study included 7634 patients (4405 in the control group and 3229 in the intervention group). The mean (SD) age was 65.4 (5.8) years; 4246 patients (55.6%) were men; 2142 (28.1%) were Black and 4625 (60.6%) were White; and 2885 (37.8%) had commercial insurance and 3950 (51.7%) had Medicare. The baseline rate of HCV screening in wedge 1 was 585 of 1560 patients (37.5% [95% CI, 35.1%-40.0%]) in hospital A and 309 of 1003 patients (30.8% [95% CI, 27.9%-33.7%]) in hospital B. The main adjusted model showed an increase of 31.8 (95% CI, 29.7-33.8) percentage points in test completion in the intervention group compared with the control group (*P* <. 001).

**Conclusions and Relevance:**

This stepped-wedge randomized clinical trial found that embedding HCV screening as a default order in the electronic health record substantially increased ordering and completion of testing in the hospital compared with a conventional interruptive alert.

**Trial Registration:**

Clinicaltrials.gov: NCT04525690

## Key Points

**Question:**

Does adding a default order to the admission order set increase screening for hepatitis C virus (HCV) among hospitalized patients?

**Findings:**

In this stepped-wedge randomized clinical trial including 7634 patients at 2 hospitals, a default order increased HCV test screening and completion compared with a conventional interruptive alert.

**Meaning:**

These findings suggest that implementing an opt-out ordering process within the electronic health record can increase HCV screening and completion rates among hospitalized patients.

## Teaser

This stepped-wedge randomized clinical trial evaluates whether a default order embedded into the admissions order set increases ordering and completion of hepatitis C virus screening among eligible hospitalized patients.

## Introduction

Hepatitis C virus (HCV) is a major cause of cirrhosis, liver transplant, and cancer in the US.^1,2^ New antiviral direct-acting agents can result in sustained virologic response in more than 95% of treated patients, which can substantially reduce the burden of disease and contribute to eradication of the virus.^3,4^ As a result, previous national guidelines since 2012 recommended birth cohort screening for individuals born between 1945 and 1965,^5,6^ and current guidelines recommend screening for all adults.^7,8^ However, despite these recommendations, HCV screening rates remain low.^9,10^ Many states, including Pennsylvania, have mandated that all health systems offer HCV screening to eligible patients who received services as an inpatient in a hospital as a complement to outpatient screening,^11,12^ but how to implement this screening and—importantly—how to ensure that offering the test results on completion of screening are unclear.

A large barrier to increasing HCV screening is human behavior—clinicians need to be aware of guidelines and order the test, and patients need to accept the offer to complete screening. New insights from the field of behavioral science identify systematic biases or heuristics that limit uptake such as status quo bias and present-time bias, which describes weighing the hassle of a screening test more heavily than the future benefits.^13,14,15,16^ The field has demonstrated how these same biases can be harnessed to increase health behaviors; for example, nudges that shift the default in ordering from opt-in to opt-out have been shown to dramatically increase generic medicine prescribing and cancer screening or to reduce unnecessary imaging.^17,18,19^ The electronic health record (EHR) offers a scalable way to implement nudges, but these need to be designed to reduce clinician effort and burden.^20^

Our own health system initially responded to the Pennsylvania mandate by implementing an interruptive EHR alert; however, uptake remained low (<20%), and the new process added clicks to the clinician workflow. Our team of clinicians, behavioral scientists, and informaticists designed an intervention to improve the existing alert. At 2 of our hospitals, we conducted a stepped-wedge randomized clinical trial to evaluate whether a default order embedded into the admissions order set could increase ordering and completion of HCV testing among eligible hospitalized patients.

## Methods

### Study Design

This stepped-wedge randomized clinical trial was approved by the institutional review board at the University of Pennsylvania, Philadelphia, which granted a waiver of informed consent because this study was an evaluation of a health system intervention associated with minimal risk that could not have been practicably performed without the waiver.^21^ The protocol and statistical analysis plan are found in Supplement 1. This trial followed the Consolidated Standards of Reporting Trials (CONSORT) reporting guideline (Supplement 1), including the checklist and diagram to track participants during the enrollment and trial procedures. Clinicians and patients were not compensated for participation.

The trial was conducted at 2 academic hospitals at the University of Pennsylvania. The stepped-wedge design involves random and sequential crossover of hospitals and allows for robust evaluation when there is a health system imperative for implementation.^22,23^ We evaluated the effect of introducing a default order for HCV antibody within the hospital admission order set on screening rates for eligible patients born between 1945 and 1965. Although the recommendation to screen all adults was finalized in March 2020,^8^ this trial was planned earlier, and there was no health system support for the new age range yet. The trial comprised three 3-month wedges, including wedge 1 (a preintervention period) from June 23 to September 20, 2020. One hospital site was randomized into the intervention during wedge 2 from September 21, 2020, to January 10, 2021, and both hospitals received the intervention during wedge 3 from January 11 to April 10, 2021. Analyses were conducted from April 26 to June 11, 2021.

### Study Population

Eligible patients were adults born between 1945 and 1965 who had an inpatient hospitalization to any clinical service at 2 hospital sites during the study period. The 2 hospitals are busy tertiary care teaching facilities located in an urban setting. Patients were excluded if they had already received HCV screening before admission (based on our health system data and data reported by clinicians in health maintenance), had previously been diagnosed with HCV, or were admitted to observation or hospice. Eligible clinicians included physicians, physician assistants, and nurse practitioners with patient encounters for hospital admissions at the sites included in the trial during the study period.

### Study Sample

Longitudinal data on patients and hospital encounters were obtained from the Clarity reporting database (Epic Systems Corporation). Patient data included information about demographics, insurance, body mass index, comorbidities, Charlson Comorbidity Index, and laboratory testing. Data on patient encounters included hospital site, admission date and type, and clinical service. As in prior work,^24^ we were not able to identify which clinician was directly exposed to the interventions.

### Randomization

The 2 hospital sites were electronically randomized to begin the intervention during 1 of the 2 intervention wedges. All investigators, statisticians, and data analysts were blinded to arm assignments until the study and analysis were completed.

### Interventions

During wedge 1, both hospital sites had a best practice alert in the EHR to prompt clinicians to consider HCV screening for eligible patients (eFigure 1 in Supplement 2). This alert was implemented 12 months before wedge 1 began. The alert stated the following: “This patient meets the criteria for hepatitis C screening (born 1945-1965). You are required to offer this screening. Please place the following order for hepatitis C antibody screen (hepatitis C antibody IgM + IgG enzyme immunoassay with reflex to hepatitis C virus PCR [polymerase chain reaction]) or provide a reason why the patient does not require this screening.” Clinicians could select either “order” or “do not order” and had the option to select from the following: “Defer until I speak with patient, patient declined, known hepatitis C, or enter a comment.” Positive HCV antibody test findings resulted in reflex viral load testing by the laboratory.

During wedge 2, the first intervention wedge, the hospital site randomized to intervention (hospital B) had the prior alert removed and an order for HCV screening embedded within the admission order set that is typically used for all patients at hospitalization (eFigure 2 in Supplement 2). For eligible patients, the order stated the following: “This patient meets criteria for hepatitis C screening (born 1945-1965). Pennsylvania law mandates offering this to all eligible patients. A positive result will be followed up by the hepatitis C linkage group to facilitate treatment and follow-up care.” The HCV screening order was prechecked, thereby creating an opt-out default to conduct the blood test during the next morning laboratory draw. Clinicians had to opt out and specify a reason (patient refused, patient recently screened, clinician declined, or other).

During wedge 2, the hospital site randomized to control (hospital A) had the prior alert continued with the text updated to match the language used in the opt-out order set (eFigure 3 in Supplement 2). The alert text read as follows: “This patient meets criteria for hepatitis C screening (born 1945-1965). Pennsylvania law mandates offering this to all eligible patients. A positive result will be followed by the hepatitis C linkage group to facilitate treatment and follow-up care.” During wedge 3, the second intervention wedge, this hospital site had the alert turned off and the HCV screening order implemented within the admission order set as described previously.

The health system sent updates on these EHR changes by email to clinicians at both hospital sites during wedge 2 and to hospital A during wedge 3. To provide coordinated and centralized care to patients screened in this intervention, the Division of Infectious Diseases at the health system created a team of clinicians (physicians [including J.T.] and nurses) who would be responsible for outreach, counseling, and care navigation to patients with a reactive HCV antibody. This hepatitis linkage team was implemented before wedge 2. This team proactively approached patients who had a positive screening assay identified through this intervention to provide test disclosure and counseling. The hepatitis linkage team followed up all reflexive quantitative viral load results and, among those with detectable viremia, offered linkage to an outpatient clinician for further evaluation and treatment.

### Outcome Measures

The primary outcome was the change in the percentage of eligible patients who received HCV screening, based on the individual-level binary outcome of whether the screening test was ordered or not. Exploratory outcomes included the change in percentage of patients who had the screening test ordered and the change in the percentage of eligible patients with a viral load positive for HCV.

### Statistical Analysis

A priori power calculations used data in early 2020 indicating that there would be approximately 1500 patients eligible for screening per site for each 3-month period and a baseline screening rate of 20%. Based on this projected sample size, we estimated we would have at least 90% power to detect a difference of 9.6 percentage points in the primary outcome of HCV screening, assuming a 2-sided α of .05 as our threshold for statistical significance. This was based on prior studies of EHR nudges and what would be a reasonable effect size to justify implementation across the health system.^17,18,25^

Both randomly assigned sites were included in the intention-to-treat analysis. We used the patient as the unit of analysis and evaluated the first encounter within each wedge period. We performed a sensitivity analysis including all patient encounters.

Similar to prior work,^19,26^ we fit generalized linear models with logit link using PROC GENMOD in SAS, version 9.4 (SAS Institute, Inc). The main model used patient-level observations and fixed effects to adjust for hospital site, wedge period, study month, and a binary indicator for intervention. To test the robustness of our model, we fit a fully adjusted model that also included age, sex, race and ethnicity, insurance, body mass index (calculated as weight in kilograms divided by height in meters squared), and Charlson Comorbidity Index score.^27^ These variables were chosen because they were available in the EHR and might be associated with chronic liver disease and ordering of HCV screening by clinicians.^28^ This analysis was performed for both tests ordered and tests completed. To obtain the adjusted difference and 95% CIs in percentage points, we used the bootstrap method, resampling patients 1000 times.^29,30^

## Results

### Patient Sample

**Figure 1.**
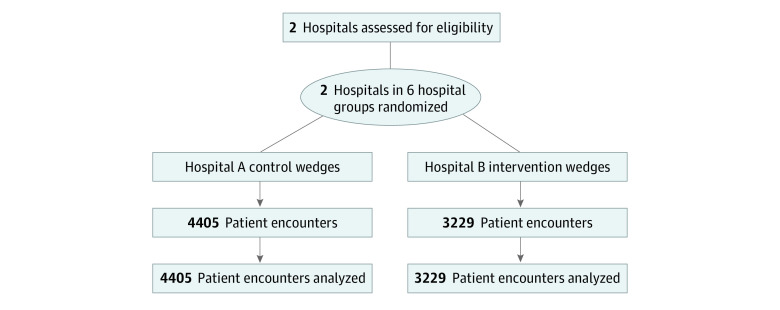
Study Flow Diagram Each hospital participated in 3 wedges: wedge 1 (a preintervention period), wedge 2 (intervention in hospital B vs control in hospital A), and wedge 3 (intervention in both hospitals).

The sample included 7634 patient encounters (Figure 1 and eFigure 4 in Supplement 2), 4405 in the control group (hospital A: wedges 1 and 2; hospital B: wedge 1) and 3229 in the intervention group (hospital A: wedge 3; hospital B: wedges 2 and 3). The mean (SD) age of the patients was 65.4 (5.8) years; 4246 patients (55.6%) were men and 3388 (44.4%) were women. With regard to race and ethnicity data, 165 patients (2.2%) were Asian; 152 (2.0%), Hispanic; 2142 (28.1%), non-Hispanic Black; 4625 (60.6%), non-Hispanic White; and 550 (7.2%), other race or ethnicity (includes American Indian, East Indian, Pacific Islander, left blank or declined to answer, other, and unknown). With regard to insurance, 2885 patients (37.8%) had commercial insurance and 3950 (51.7%) had Medicare (Table 1). Patients in the control and intervention groups had similar characteristics, but the intervention group had more Black patients (1034 of 3229 [32.0%] vs 1108 of 4405 [25.2%]) owing to differences in hospital patient population.

**Table 1.** Patient Characteristics

Characteristic	Hospital by condition^a^					
	Control			Intervention		
	Hospital A (n = 3402)	Hospital B (n = 1003)	Both hospitals (n = 4405)	Hospital A (n = 1364)	Hospital B (n = 1865)	Both hospitals (n = 3229)
Sociodemographic						
Age, mean (SD), y	65.4 (5.8)	65.4 (5.7)	65.4 (5.8)	65.6 (5.9)	65.4 (5.9)	65.5 (5.9)
Sex						
Men	1901 (55.9)	549 (54.7)	2450 (55.6)	747 (54.8)	1049 (56.2)	1796 (55.6)
Women	1501 (44.1)	454 (45.3)	1955 (44.4)	617 (45.2)	816 (43.7)	1433 (44.4)
Race and ethnicity						
Asian	93 (2.7)	15 (1.5)	108 (2.5)	34 (2.5)	23 (1.2)	57 (1.8)
Hispanic	69 (2.0)	19 (1.9)	88 (2.0)	29 (2.1)	35 (1.9)	64 (2.0)
Non-Hispanic Black	724 (21.3)	384 (38.3)	1108 (25.1)	296 (21.7)	738 (39.6)	1034 (32.0)
Non-Hispanic White	2276 (66.9)	501 (49.9)	2777 (63.0)	914 (67.0)	934 (50.1)	1848 (57.2)
Other^b^	240 (7.1)	84 (8.4)	324 (7.3)	91 (6.7)	135 (7.2)	226 (7.0)
Insurance						
Commercial	1367 (40.2)	318 (31.7)	1685 (38.3)	573 (42.0)	627 (33.6)	1200 (37.2)
Medicare	1754 (51.5)	542 (54.0)	2296 (52.1)	684 (50.1)	970 (52.0)	1654 (51.2)
Medicaid	281 (8.3)	143 (14.3)	424 (9.6)	107 (7.8)	268 (14.4)	375 (11.6)
Clinical						
Body mass index, mean (SD)^c^	28.8 (6.8)	31 (8.7)	29.3 (7.3)	29.3 (7.1)	30.4 (8.3)	29.9 (7.8)
Charlson Comorbidity Index score, median (IQR)	2 (1-4)	2 (0-3)	2 (1-4)	2 (1-4)	1 (0-3)	2 (0-3)

^a^ Unless otherwise indicated, data are expressed as number (%) of patients. Percentages are rounded and may not total 100.

^b^ Includes American Indian, East Indian, Pacific Islander, left blank or declined to answer, other, and unknown.

^c^ Data were missing for 110 patients. Calculated as weight in kilograms divided by height in meters squared.

### Test Ordering

**Figure 2.**
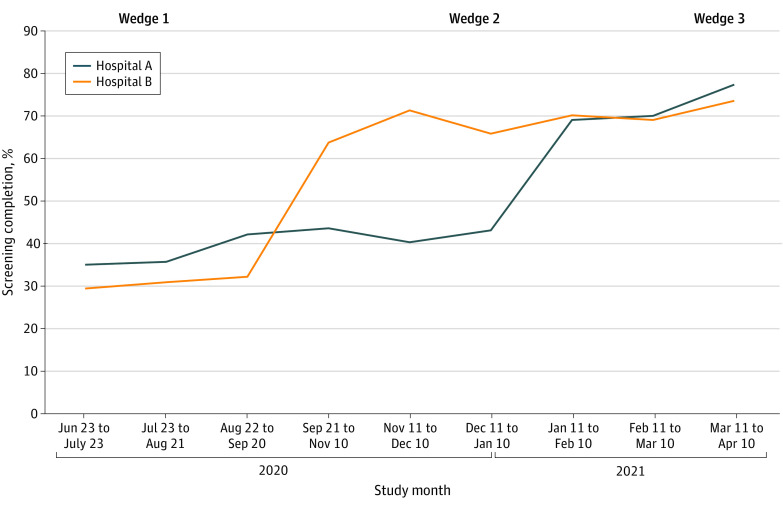
Hepatitis C Virus Screening Completion by Hospital Site and Study Month Wedge 1 was a preintervention period. Hospital A (control hospital) received the intervention during wedge 3; hospital B received the intervention during wedges 2 and 3.

The baseline rate of HCV test ordering (exploratory outcome) during wedge 1 was 647 of 1560 (41.5% [95% CI, 39.1%-43.9%]) in hospital A and 346 of 1003 (34.5% [95% CI, 31.6%-37.4%]) in hospital B (Table 2 and Figure 2). Among patients in both hospitals, 1868 of 4405 patients in the control group (42.4%) had the test ordered compared with 2599 of 3229 patients in the intervention group (80.5%). The main adjusted model accounting for hospital site, wedge period, and study month showed an increase of 38.1 (95% CI, 36.1-40.0) percentage points in ordering in the intervention group compared with the control groups (*P* < .001) (Table 3). The fully adjusted model that also included patient age, sex, race and ethnicity, insurance, body mass index, and Charlson Comorbidity Index score showed an increase of 38.1 (95% CI, 36.1-40.0) percentage points in test ordering (*P* <. 001).

**Table 2.** Hepatitis C Virus Screening Test Ordering and Completion

Hospital	Period, No. screened/total No. (%)			
	Wedge 1 (6/23/2020 to 9/20/2020)	Wedge 2 (9/21/2020 to 1/10/2021)	Wedge 3 (1/11/2021 to 4/10/2021)	All
Test ordered				
Hospital A	647/1560 (41.5)	875/1842 (47.5)	1152/1364 (84.5)	2674/4766 (56.1)
Hospital B	346/1003 (34.5)	811/1055 (76.9)	636/810 (78.5)	1793/2868 (62.5)
Both	993/2563 (38.7)	1686/2897 (58.2)	1788/2174 (82.2)	4467/7634 (58.5)
Test completed				
Hospital A	585/1560 (37.5)	785/1842 (42.6)	983/1364 (72.1)	2353/4766 (49.4)
Hospital B	309/1003 (30.8)	698/1055 (66.2)	576/810 (71.1)	1583/2868 (55.2)
Both	894/2563 (34.9)	1483/2897 (51.2)	1559/2174 (71.7)	3936/7634 (51.5)

**Table 3.** Effect of Default Intervention on Hepatitis C Virus Screening

	Patient group, No. screened/total No. (%)		Intervention vs control		*P* value	Information criterion	
	Control	Intervention	OR (95% CI)	Adjusted difference, % (95% CI)^a^		Akaike	Bayesian
Test ordered							
Main adjusted model^b^	1868/4405 (42.4)	2599/3229 (80.5)	5.11 (4.12-6.34)	38.1 (36.1-40.0)	<.001	9136.7	9240.8
Fully adjusted model^c^	NA	NA	5.63 (4.51-7.02)	38.1 (36.1-40.0)	<.001	8893.2	9073.6
Test completed							
Main adjusted model^b^	1679/4405 (38.1)	2257/3229 (69.9)	3.18 (2.59-3.89)	31.8 (29.7-33.8)	<.001	9771.3	9875.4
Fully adjusted model^c^	NA	NA	3.41 (2.78-4.19)	31.8 (29.7-33.8)	<.001	9563.0	9743.4

Abbreviations: NA, not applicable; OR, odd ratio.

^a^ To obtain the adjusted difference and 95% CI in percentage points, we used the bootstrap method, resampling patients 1000 times.

^b^ Main adjusted model includes hospital site, wedge period, and study month.

^c^ Fully adjusted model also includes age, sex, race and ethnicity, insurance, body mass index (calculated as weight in kilograms divided by height in meters squared), and Charlson Comorbidity Index score.

### Test Completion

The baseline rate of HCV screening (primary outcome) during wedge 1 was 585 of 1560 (37.5% [95% CI, 35.1%-40.0%]) in hospital A and 309 of 1003 (30.8% [95% CI, 27.9%-33.7%]) in hospital B. Among the patients in both hospitals, 1679 of 4405 patients in the control group (38.1%) had the test completed compared with 2257 of 3229 patients in the intervention group (69.9%). The main adjusted model showed an increase of 31.8 (95% CI, 29.7-33.8) percentage points in test completion in the intervention group compared with the control group (*P* < .001) (Table 3). The fully adjusted model showed an increase of 31.8 (95% CI, 29.7-33.8) percentage points in test completion (*P* < .001). We conducted main adjusted and fully adjusted analyses including all visits (not just the first visit by a patient) and found similar results for the main outcomes.

### Test Results and Follow-up

Of all patients included in the study, 29 (0.7% [95% CI, 0.4%-0.9%]) had a positive viral load in the control group and 34 (1.1% [95% CI, 0.7%-1.4%]) had a positive viral load in the intervention group. The resultant increase in identifying patients with positive viremia (exploratory outcome) was 0.4% (95% CI, 0.003%-0.8%; *P* = .06).

During the study period, 49 patients with a positive viral load were identified by the hepatitis linkage team through automated data reports, of whom 46 (93.9%) were approached for counseling and linkage. Of these, 39 patients (84.8%) were linked to a treating clinician, 27 within our own health system. Of the 27 patients linked at our own health system, 16 (59.3%) were prescribed direct-acting antiviral medications for HCV, of which 12 (75.0%) were filled by our health system pharmacy.

## Discussion

In this stepped-wedge randomized clinical trial, we found that embedding HCV screening as a default order for screening-eligible patients in the EHR increased ordering and completion of testing in the hospital compared with a conventional interruptive alert. Because this was an EHR-based intervention, there was minimal incremental cost to clinicians or the health system after implementation, and it potentially saved clinician time by reducing clicks. In addition, although it was an exploratory outcome and not statistically significant, the intervention resulted in a 0.4% increase in identification of patients with a positive viral load for HCV, representing approximately 13 additional patients.

There are a few reasons that this EHR default may have been effective at increasing test completion. First, the intervention shifted HCV screening from opt-in to opt-out. For most screening tests, the default is not to order the test, and it requires decision-making to actively choose the test. In this case, the default was that HCV screening was going to be ordered, and it required the clinician to actively click the order to opt out. Second, it embedded HCV screening into the routine EHR workflow.^31^ Clinicians were already ordering an admission order set, so they did not have to find the order in another part of the EHR. This builds on prior work that showed that default orders can increase uptake of high-value and appropriate medication prescribing.^31,32,33^ Third, the default order reduced the number of clicks required by the clinician to order HCV testing, making the right choice the easy choice.^31,34^ The default order still allowed clinicians to easily opt out in cases where it was not appropriate, which is supported by approximately 30% of patients not receiving HCV testing after the intervention. It is not clear why this many patients remained unscreened, but it may have been because the patient declined, the clinician deferred decision-making, there was screening outside the health system, or the patient was discharged before the laboratory samples could be drawn. When a nudge has limited effectiveness for an important clinical activity, a stronger intervention—a “shove”—may be needed.^35^

These findings add to the literature of EHR-based interventions to boost HCV screening uptake. Yartel et al^25^ conducted a cluster randomized trial at an academic medical center and found that a conventional interruptive best-practice alert to the medical assistant and clinician in a primary care setting resulted in an increase in screening completion from 3.6% in the control group to 30.9% in the intervention group. Another trial in the primary care setting at our own health system^36^ showed that bulk ordering with opt-out framing increased HCV screening from 19.2% to 43.1%. Interestingly, in the outpatient setting, only 0.4% of screening tests completed were positive for viral load, whereas 1.6% were positive in the previous study, suggesting that inpatients may have a higher risk of HCV.

### Strengths and Limitations

Strengths of this study include its prospective design and randomization at the hospital level. The stepped-wedge design allowed this to be implemented sequentially and pragmatically at both hospitals while still allowing for evaluation of effectiveness. In doing so, we were also able to avoid contamination of the effect between clinicians at the same delivery setting. It was conducted in close partnership with clinical operations in a naturalized setting with minimal exclusions. The setting was a busy urban academic hospital with many different types of clinicians, which may translate to other care delivery environments.

There were some limitations to this study. There were differences in the hospital characteristics, as seen by the slightly different patient populations, although they were in the same health system and city. We also saw an increase in screening rate at the control hospital wedges over time, likely from other concurrent interventions or familiarity with the hepatitis linkage team program. However, the prospective randomization and stepped-wedge design where both sites eventually received the intervention likely controlled for these factors. Although we were able to pull data from our health system on prior screening and some outside data, we may have missed testing at outside systems, resulting in possible overscreening. Some clinicians may have worked at both hospitals, resulting in contamination, but that number is low and would have biased toward a null effect. In addition, the experiences from this academic environment may not translate to other hospital settings or populations across the country.

## Conclusions

This stepped-wedge randomized clinical trial found that default order options increased completion rates of HCV screening. These results could translate to expanded HCV screening among all adults and benefit other important clinical quality initiatives.

## Notes

Supplement 1.
Trial Protocol

Supplement 2. **eFigure 1.** Best Practice Alert in Place Before the Study Period **eFigure 2.** Opt-out Hepatitis C Viral Screening Within the Admissions Order Set **eFigure 3.** Best Practice Alert for the Control Hospital Site During the First Intervention Wedge **eFigure 4.** Stepped-Wedge Design

Supplement 3.
Data Sharing Statement
