# Supplementary material for: Effect of a Default Order vs an Alert in the Electronic Health Record on Hepatitis C Virus Screening Among Hospitalized Patients: A Stepped-Wedge Randomized Clinical Trial
Source: JAMA Netw Open. 2022 Mar 17;5(3):e222427. doi: 10.1001/jamanetworkopen.2022.2427 (PMC8931559; doi:10.1001/jamanetworkopen.2022.2427)
Supplement: Supplement 1. — Trial Protocol [file jamanetwopen-e222427-s001.pdf]

Improving Inpatient Screening for Hepatitis C

A Stepped-Wedge Randomized Clinical Trial

Study Protocol

April 2020

Outline

1. Abstract

2. Overall objectives

3. Aims

    3.1 Primary outcome

    3.2 Exploratory outcomes

4. Background

5. Study design

    5.1 Design

    5.2 Study duration

    5.3 Target population

    5.4 Accrual

    5.5 Key inclusion criteria

    5.6 Key exclusion criteria

6. Subject recruitment

7. Subject compensation

8. Study procedures

    8.1 Consent

    8.2 Procedures

9. Analysis plan

10. Investigators

11. Human research protection

    11.1 Data confidentiality

    11.2 Subject confidentiality

    11.3 Subject privacy

11.4 Data disclosure

11.5 Data safety and monitoring

11.6 Risk/benefit

11.6.1 Potential study risks

11.6.2 Potential study benefits

11.6.3 Risk/benefit assessment

1. Abstract

The hepatitis C virus (HCV) is the leading cause of liver transplant and hepatocellular carcinoma in the United States, but direct-acting antiviral medications are now available and can cure the disease in over 95% of those that are treated. The Centers for Disease Control and Prevention (CDC) estimate that 75% of all chronic HCV infections in the United States are among adults born between 1945 and 1965. The CDC and US Preventive Services Task Force (USPTF) therefore recommends birth cohort screening for all adults born in this time period. In 2016, the Commonwealth of Pennsylvania signed into law a requirement that all hospitalized patients born during this time period be offered HCV screening. At two Penn Medicine hospitals, about 1000 eligible patients are admitted each month and despite an EHR alert that prompts ordering for eligible patients less than 20% are screened. In this study, we will conduct a stepped-wedge cluster randomized clinical trial to test the effect of defaulting HCV screening into the admission order set to improve screening rates.

2. Overall objectives

The objective of the study is to evaluate the effect of a health system initiative setting defaults in the electronic health record admission order set to nudge inpatient HCV screening.

3. Aims

3.1 Primary outcome

The primary outcome measure is the change in percentage of eligible patients that receive HCV antibody screening.

3.2 Exploratory outcomes

The exploratory outcomes include the change in the percentage of eligible patients who are viral load positive for HCV and who are HCV antibody positive. If data is available, we will explore the percentage of patients that have positive HCV testing who receive either linkage to care and/or HCV treatment

4. Background

The hepatitis C virus (HCV) is the leading cause of liver transplant and hepatocellular carcinoma, but direct-acting antiviral medications are now available and can cure the disease in over 95% of those who are treated. The Centers for Disease Control and Prevention (CDC) estimate that 75% of all chronic HCV infections in the United States are among adults born between 1945 and 1965. The CDC and USPTF therefore recommends screening for all adults born in this time period. In 2016, the Commonwealth of Pennsylvania signed into law a requirement that all hospitalized patients born during this time period be offered HCV screening. At two Penn Medicine hospitals, about 1000 eligible patients are admitted each month and

despite an EHR alert that prompts ordering for eligible patients (implemented May 14, 2019) less than 20% are screened.

Defaults are the path of least resistance and often seen as an implicit recommendation. They are often fit better into clinician workflow because they do not require alerts or action to be taken. They are most appropriate when eligible patients can be accurately identified and defaults can be aligned with guidelines, in this case from the CDC, USPTF and Commonwealth of Pennsylvania. In prior work at Penn Medicine, we have used changes in defaults to increase generic prescribing, increase colorectal cancer screening, and decrease unnecessary imaging among palliative cancer patients.

5. Study design

5.1 Design

This study will use a stepped-wedge cluster randomized clinical trial to evaluate a health system initiative to improve inpatient HCV screening. We will randomly assign either the Hospital of the University of Pennsylvania (HUP) or Penn Presbyterian Medical Center (PPMC) to have the default intervention implemented in the admission order set within the electronic health record (EHR). Clinicians will have the opportunity to opt out and not order the screening test. There will be 3 wedges, each 3 months in duration, including a 3-month pre-intervention period, a 3-month period where only the site randomly selected to go first will have the intervention implemented, and then a 3-month period where both sites will have the intervention implemented. We will also look retrospectively at HCV screening rates before and after the EHR alert was implemented on May 14, 2019.

5.2 Study duration

The study is expected to begin in September 2020 and take one year to complete.

5.3 Target population

Adults born between 1945 and 1965 and admitted to either HUP or PPMC who have not already received HCV screening.

5.4 Accrual

Based on data from early 2020, we expect about 1500 patients per site over a three month period. Therefore, we estimate that for HCV screening, we will have 90% power to detect a 9.6-percentage point difference and 80% power to detect an 8.2 percentage point difference. This assumes a baseline rate of 20% and a 2-sided α of 0.05 as our threshold for statistical significance. For detecting viral load positive patients, we estimate that we will have 90% power to detect a 3.7-percentage point increase and 80% power to detect a 2.9-percentage point

increase. This assumes a baseline rate of 0.5% and a 2-sided α of 0.05 as our threshold for statistical significance.

5.5 Key inclusion criteria

Patients must meet the following criteria to be eligible for the study:

1) Be admitted to HUP or PPMC; 2) Born between 1945 and 1965

5.6 Key exclusion criteria

Patients will be excluded if they have already received HCV screening or have been previously diagnosed with HCV.

6. Subject recruitment

This is a pragmatic study of a health system intervention. Clinicians and patients will be exposed to the intervention as a part of their usual care during inpatient hospital admission.

7. Subject compensation

No compensation will be offered in this study.

8. Study procedures

8.1 Consent

A waiver of informed consent is requested. This is a health system initiative that will be implemented. The study is to evaluate that initiative. Therefore, physicians and their patients will not be consented as this is the standard of practice per the health system initiative. Without a waiver of the consent, the initiative would still be implemented by the health system, but the study would be infeasible. There are several additional reasons why we feel a waiver of consent should be granted. First, it is not feasible to consent every patient and physician and as mentioned this initiative would occur with or without the study of it. Second, if members of the control group were consented, they would know they were being studied and this could change their behavior. This could potentially disrupt the design of the study and making interpretation of the findings challenging. Third, physicians are not being forced to order HCV screening for their patients. Instead, they are being reminded of evidence-based CDC and USPSTF guidelines and Pennsylvania Law and offered an opportunity to order HCV screening. This is no different than standard of care in which a physician would review the same information and decide to pursue HCV screening or not.

8.2 Procedures

Data on clinicians and their patients at the University of Pennsylvania Health System will be obtained from Penn Data Store and Clarity (Epic's data reporting database). Physician data

includes demographic information (age, race, gender, type of medical degree, etc.) and may be also obtained from publicly available databases or websites online. Patient information includes demographic information, information about comorbid conditions (including diabetes, hypertension, and chronic liver disease, and comorbid conditions needed to calculate the Charleston Comorbidity Index and laboratory test results (HCV antibody and viral load) and medications to treat HCV.

9. Analysis plan

All analyses will be conducted using intention-to-treat using the patient as the unit of analysis. All hypothesis tests will use a two-sided alpha of 0.05 as our threshold for statistical significance.

The primary outcome measure will use a binary indicator representing the presences of HCV screening or not for each patient. In the main adjusted analysis, we will fit models using generalized estimating equations using group (HUP or PPMC), period (3-month increment) fixed effects, and a variable for intervention (default implemented or not) and adjusting for temporal trends.

To test the robustness of our findings, we will perform sensitivity analyses that adjusts for available patient characteristics and comorbidities such as demographics and the Charlson Comorbidity Index.

10. Investigators

Mitesh Patel, MD, MBA is Multiple Principal Investigator (PI), Assistant Professor of Medicine and Health Care Management at the Perelman School of Medicine and The Wharton School at the University of Pennsylvania, a general internist, and Director of the Penn Medicine Nudge Unit. He has extensive experience leading clinical trials deployed within the health system.

Shivan Mehta, MD, MBA is the Multiple Principal Investigator (PI) is an Assistant Professor of Medicine at the Perelman School of Medicine, a gastroenterologist, and Associate Chief Innovation Officer for Penn Medicine. He has extensive experience leading clinical trials deployed within the health system.

11. Human research protection

11.1 Data confidentiality

Computer-based files will only be made available to personnel involved in the study through the use of access privileges and passwords. Wherever feasible, identifiers will be removed from study-related information. Precautions are already in place to ensure the data are secure by using passwords and HIPAA-compliant encryption.

11.2 Subject confidentiality

Data on physicians and patients will be obtained from Epic and Penn Data Store. Any information that is obtained will be used for research purposes only. Information on patients will only be disclosed within the study team. All study staff will be reminded of the confidential nature of the data collected and contained in these databases.

Data will be stored, managed, and analyzed on a secure, encrypted server behind the University of Pennsylvania Health System (UPHS) firewall. This server was created for projects conducted by the Penn Medicine Nudge Unit related to physician and patient behavior at UPHS. All study personnel that will use this data are listed on the IRB application and have completed training in HIPAA standards and the CITI human subjects research. Data access will be password protected. Whenever possible, data will be deidentified for analysis.

11.3 Subject privacy

All efforts will be made by study staff to ensure subject privacy. Data will be evaluated in a de-identified manner whenever possible.

11.4 Data disclosure

Information on physicians and patients will not be disclosed to anyone outside of the study team.

11.5 Data safety and monitoring

The investigators will provide oversight for the study evaluation of this health system initiative. Clinicians will follow their standards of care to manage patients admitted to the hospital.

11.6 Risk/benefit

11.6.1 Potential study risks

The potential risks associated with this study are minimal. Breach of data is a potential risk that will be mitigated by using HIPAA compliant and secure data platforms for the intervention (Penn Medicine servers) and evaluation (Nudge Unit server). HCV screening is conducted as standard of care and involves a blood draw.

11.6.2 Potential study benefits

HCV screening can identify patients with the infection who could be treated.

11.6.3 Risk/benefit assessment

The risk/benefit ratio is highly favorable given the potential benefit from patients with positive HCV screening who could be treated and that efforts have been put into place to minimize the risk of breach of data.
