# Supplementary material for: Effect of a Default Order vs an Alert in the Electronic Health Record on Hepatitis C Virus Screening Among Hospitalized Patients: A Stepped-Wedge Randomized Clinical Trial
Source: JAMA Netw Open. 2022 Mar 17;5(3):e222427. doi: 10.1001/jamanetworkopen.2022.2427 (PMC8931559; doi:10.1001/jamanetworkopen.2022.2427)
Supplement: Supplement 2. — eFigure 1. Best Practice Alert in Place Before the Study Period eFigure 2. Opt-out Hepatitis C Viral Screening Within the Admissions Order Set eFigure 3. Best Practice Alert for the Control Hospital Site During the First Intervention Wedge eFigure 4. Stepped-Wedge Design [file jamanetwopen-e222427-s002.pdf]

Supplemental Online Content

Mehta SJ, Torgersen J, Small DS, et al. Effect of a default order vs an alert in the electronic health record on hepatitis C virus screening among hospitalized patients: a stepped-wedge randomized clinical trial. *JAMA Netw Open*. 2022;5(3):e222427.
doi:10.1001/jamanetworkopen.2022.2427

eFigure 1. Best Practice Alert in Place Before the Study Period

eFigure 2. Opt-out Hepatitis C Viral Screening Within the Admissions Order Set

eFigure 3. Best Practice Alert for the Control Hospital Site During the First Intervention Wedge

eFigure 4. Stepped-Wedge Design

This supplemental material has been provided by the authors to give readers additional information about their work.

© 2022 Mehta SJ et al. *JAMA Network Open*.

eFigure 1. Best Practice Alert in Place Before the Study Period

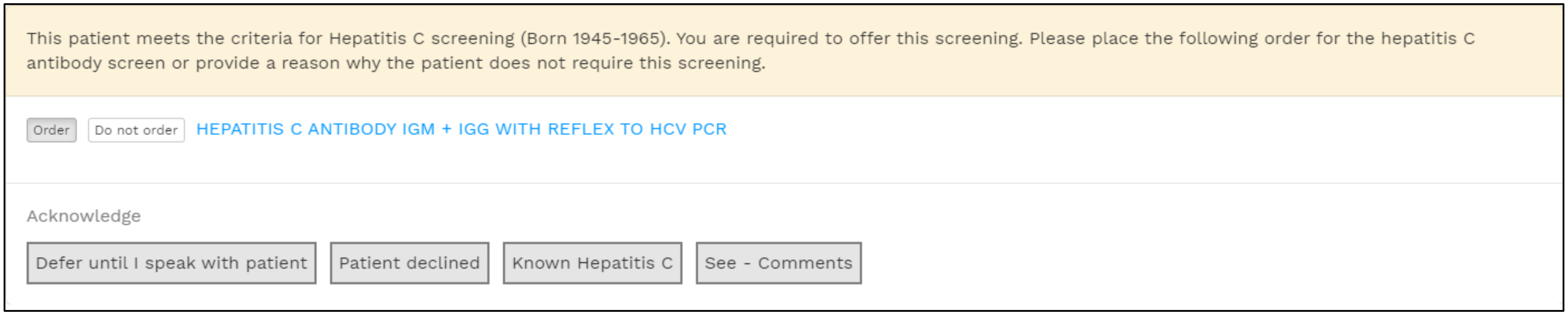

© 2022 Mehta SJ et al. *JAMA Network Open*.

eFigure 2. Opt-out Hepatitis C Viral Screening Within the Admissions Order Set

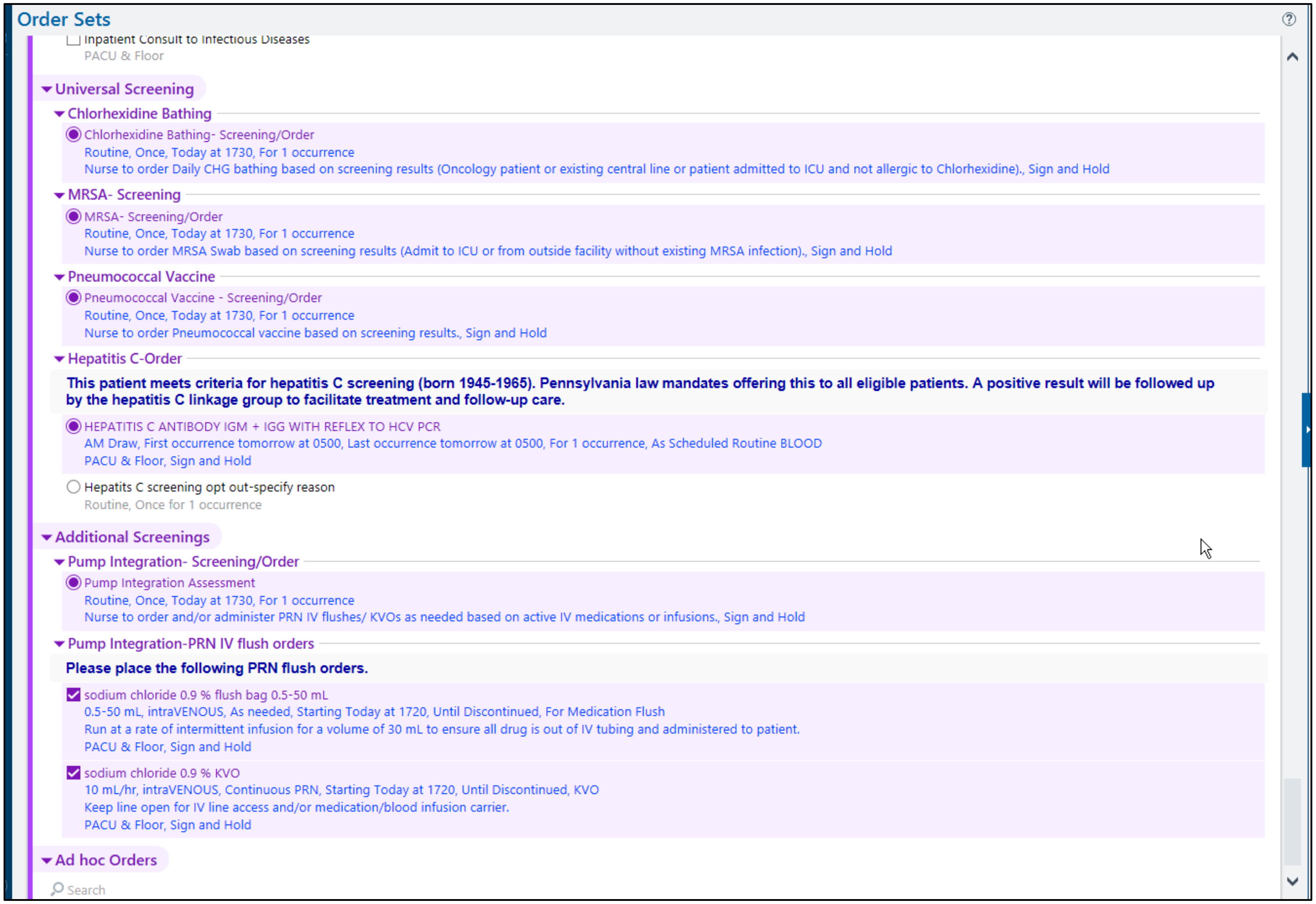

© 2022 American Medical Association. All rights reserved.

eFigure 3. Best Practice Alert for the Control Hospital Site During the First Intervention Wedge

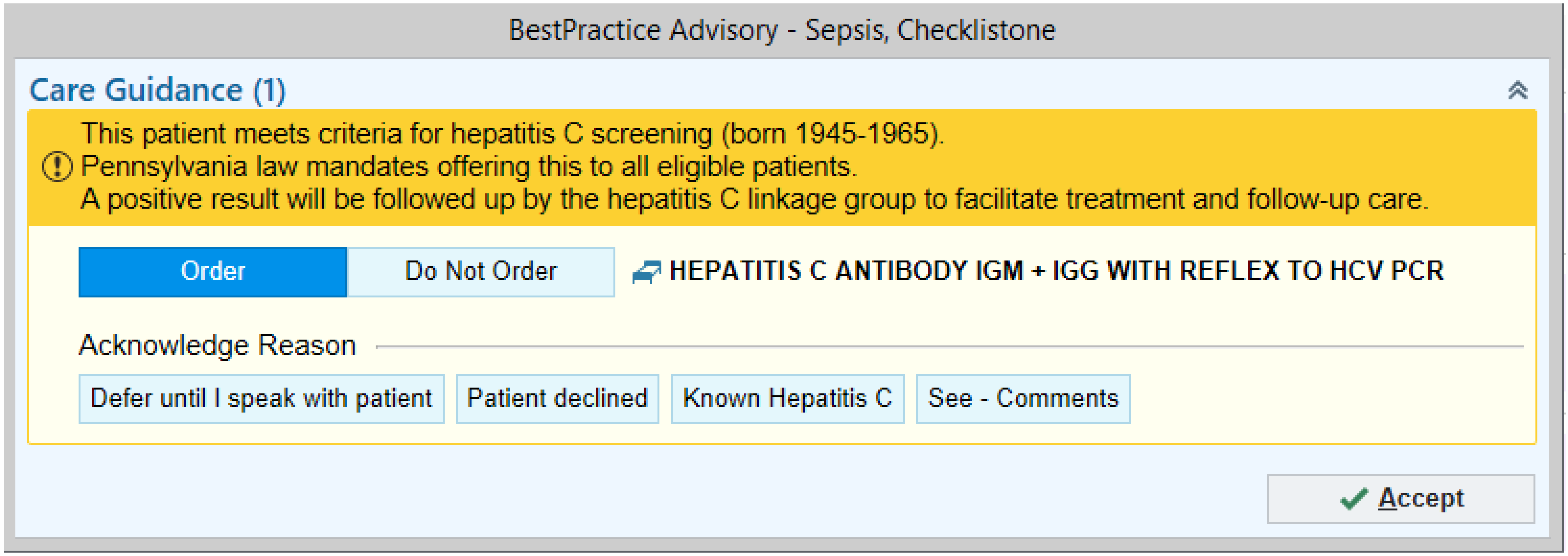

© 2022 Mehta SJ et al. *JAMA Network Open*.

eFigure 4. Stepped-Wedge Design

Cluster	Pre-Intervention (6/23/2020-9/20/2020)	Intervention 1 (9/21/2020-1/10/2021)	Intervention 2 (1/11/2021-4/10/2021)
Hospital A	1560	1842	1364
Hospital B	1003	1055	810

Intervention (dark blue)

Control (light blue)

© 2022 Mehta SJ et al. *JAMA Network Open*.
