# Supplementary material for: Effect of a Default Order vs an Alert in the Electronic Health Record on Hepatitis C Virus Screening Among Hospitalized Patients: A Stepped-Wedge Randomized Clinical Trial
Source: JAMA Netw Open. 2022 Mar 17;5(3):e222427. doi: 10.1001/jamanetworkopen.2022.2427 (PMC8931559; doi:10.1001/jamanetworkopen.2022.2427)
Supplement: Supplement 3. — Data Sharing Statement [file jamanetwopen-e222427-s003.pdf]

Data Sharing Statement

Mehta. Effect of a Default Order vs an Alert in the Electronic Health Record on Hepatitis C Virus Screening Among Hospitalized Patients. *JAMA Netw Open*. Published March 17, 2022. doi:10.1001/jamanetworkopen.2022.2427

Data
Data available: No

Additional Information
Explanation for why data not available: We do not have permission from the IRB or the health system.
